# Endothelial cell junctional adhesion molecule C plays a key role in the development of tumors in a murine model of ovarian cancer

**DOI:** 10.1096/fj.13-230441

**Published:** 2013-10

**Authors:** David A. Leinster, Bartomeu Colom, James R. Whiteford, Darren P. Ennis, Michelle Lockley, Iain A. McNeish, Michel Aurrand-Lions, Triantafyllos Chavakis, Beat A. Imhof, Frances R. Balkwill, Sussan Nourshargh

**Affiliations:** * William Harvey Research Institute and; † Barts Cancer Institute, Barts and The London School of Medicine and Dentistry, Queen Mary University of London, London, UK;; ‡ Centre de Recherche en Cancérologie de Marseille, Institut National de la Santé et de la Recherche Médicale (INSERM) Unité Mixte de Recherche (UMR) 1068, Aix-Marseille Université, Institut Paoli-Calmettes, Marseille, France;; § Dresden University of Technology, Dresden, Germany; and; ‖ Centre Médical Universitaire, Geneva, Switzerland

**Keywords:** pericytes, angiogenesis, vascular development, immune cell infiltrate

## Abstract

Junctional adhesion molecule C (JAM-C) is a transmembrane protein with significant roles in regulation of endothelial cell (EC) functions, including immune cell recruitment and angiogenesis. As these responses are important in promoting tumor growth, the role of EC JAM-C in tumor development was investigated using the ID8 syngeneic model of ovarian cancer. Within 10–15 wk, intraperitoneally injected ID8 cells form multiple tumor deposits and ascites that resemble human high-grade serous ovarian cancer. Compared to wild-type mice, survival in this model was increased in EC JAM-C knockouts (KOs; 88 *vs.* 96 d, *P*=0.04) and reduced in EC JAM-C transgenics (88 *vs.* 78.5 d, *P*=0.03), mice deficient in or overexpressing EC JAM-C, respectively. While tumor growth was significantly reduced in EC JAM-C KOs (87% inhibition at 10 wk, *P*<0.0005), this was not associated with alterations in tumor vessel density or immune cell infiltration. However, tumor microvessels from EC JAM-C-deficient mice exhibited reduced pericyte coverage and increased vascular leakage, suggesting a role for EC JAM-C in the development of functional tumor vessels. These findings provide evidence for a role for EC JAM-C in tumor growth and aggressiveness as well as recruitment of pericytes to newly formed blood vessels in a model of ovarian cancer.—Leinster, D. A., Colom, B., Whiteford, J. R., Ennis, D. P., Lockley, M., McNeish, I. A., Aurrand-Lions, M., Chavakis, T., Imhof, B. A., Balkwill, F. R., Nourshargh, S. Endothelial cell junctional adhesion molecule C plays a key role in the development of tumors in a murine model of ovarian cancer.

Junctional adhesion molecule C (JAM-C) is a member of the immunoglobulin superfamily and is expressed by a diverse range of cell types including endothelial cells (ECs), epithelial cells, smooth muscle cells, and Schwann cells, and in humans it is also found on platelets and certain leukocyte subtypes (1–7). Its broad expression profile accounts for the wide range of functional responses linked to JAM-C including regulation of vascular permeability, leukocyte migration, angiogenesis, and nerve morphology and function (2, 6, 8–11). Through the use of JAM-C-deficient mice and/or blocking antibodies, the role of JAM-C has been investigated in numerous disease models such as models of arthritis (12), peritonitis (13), acute pancreatitis (14), ischemia-reperfusion injury (11), and pulmonary inflammation (15, 16). Furthermore, in humans, vascular expression of JAM-C appears to be enhanced under certain inflammatory disease conditions such as atherosclerosis and rheumatoid arthritis (3, 12). Despite the above, many aspects of the functional role of EC JAM-C remain unknown, and its role in the development and progression of chronic inflammatory diseases is unclear. As EC JAM-C plays a significant role in regulating leukocyte-EC interactions and angiogenesis, it may also be involved in cancer and tumor development. Indeed, a recent study demonstrated inhibition of melanoma lung metastasis in both full and EC-specific JAM-C-knockout (KO) mice (17). Furthermore, an anti-JAM-C blocking mAb has been shown to suppress the growth of glioma and Lewis lung carcinoma tumors in mice (18, 19). Despite the above studies, little is known about the role of EC JAM-C in cancer, an issue that is investigated here in the context of a murine model of ovarian cancer.

The term ovarian cancer refers to a number of malignancies of different tissue origin that spread throughout the peritoneal cavity (20, 21). The commonest subtype, ovarian high-grade serous cancer (HGSC), is usually diagnosed after the disease has disseminated throughout the peritoneum, and as a result, 5-yr survival statistics are poor (UK 43%; ref.22). Current treatment for HGSC is surgical debulking and platinum/taxane-based therapeutics, highlighting the need for better understanding of the pathogenesis of this condition and development of novel and more effective therapeutics. Investigations of HGSC have been hampered by the lack of reliable genetic experimental models (23). However, we recently reported that intraperitoneal injection of ID8 cells into mice leads to multiple tumor deposits with a tumor microenvironment that recapitulates human HGSC with substantial leukocyte infiltrates and functional tumor vasculature (24–26). The role of EC JAM-C in these events was investigated using conditional mice with reduced expression of EC JAM-C (EC JAM-C KO; ref. 27) and transgenic mice with overexpression of EC JAM-C (EC JAM-C Tg; 15). Results show that deletion of endothelial JAM-C leads to enhanced survival of mice as compared to wild types (WTs), whereas increased expression of EC JAM-C leads to reduced survival times. EC JAM-C-KO mice also showed slower tumor growth, as well as reduced pericyte coverage and compromised functionality of tumor blood vessels.

## MATERIALS AND METHODS

### Animals

EC JAM-C-KO (*Tie2Cre;JAM-C*^*flox/flox*^; 129/C57BL/6 background; refs. 17, 27) and EC JAM-C-Tg (15) mice (C57BL/6 background) were generated as previously detailed. Littermate WTs were used as controls. Animal experiments were conducted in accordance with UK legislation.

### Human tissue samples

Human omentum and primary tumor samples were collected after surgery on patients with ovarian cancer at St. Bartholomew's Hospital (London, UK). Samples were taken from patients who had given prior consent under Research Ethical Committee Project 10/H0304/14 and stored within the Barts Gynae Tissue Bank.

### Cell line and ID8 peritoneal tumor model

ID8 malignant ovarian surface epithelial cells (provided by K. Roby, University of Kansas, Kansas City, KS, USA) were cultured in DMEM medium with 4% FCS (PAA, Yeovil, UK), Insulin Transferrin Sodium Selenite Supplement (Sigma-Aldrich, Dorset, UK), and penicillin/streptomycin (PAA). For some experiments, cells were infected with luciferase constructs (pHRSIN-CSGW-dNotI; kindly provided by Dr. Y. Ikeda, Mayo Clinic, Rochester, MN, USA) as described previously (28).

ID8 cells were implanted by intraperitoneal injection to form peritoneal tumor nodules over a 10- to 15-wk period. Welfare of the animals was monitored, and mice were euthanized as per UK Coordinating Committee on Cancer Research (UKCCR) guidelines (29). Tumor samples were removed *post mortem* and either frozen in optimum cutting temperature (OCT) compound (Fisher Scientific, Loughborough, UK) for sectioning or snap-frozen in liquid nitrogen for protein extraction. Ascitic fluid was collected and centrifuged, and supernatants were stored at −80°C.

### Monitoring of tumor growth

Tumor growth was investigated *in vivo via* bioluminescent imaging, as described previously (28). Briefly, animals implanted with luciferase-expressing ID8 cells were injected with d-luciferin, and the bioluminescence intensity was measured using an IVIS bioluminescent system (IVIS; Xenogen, Alameda, CA, USA). Images were quantified using Living Imaging software (Xenogen) from a region of interest of the animal's ventral surface.

### Aortic ring *ex vivo* angiogenesis model

The aortic ring assay was used as an *ex vivo* model of angiogenesis (30). Thoracic aortas from 6- to 10-wk-old mice were dissected, and the fatty tissue and branches were removed. Aortas were cut into thin sections and cultured in Opti-Mem (Invitrogen, Paisley, UK) medium overnight at 37°C without serum. The rings were embedded in 1 mg/ml type I collagen (Millipore, Watford, UK) in E4 medium (PAA) in 48-well dishes. After polymerization of the collagen, Opti-Mem medium with 1% heat inactivated serum and 30 ng/ml VEGF was added. Bright-field images were acquired using an Olympus IX81 inverted microscope (Olympus Medical, Tokyo, Japan). After 2 wk, the ring tissues were fixed with 4% PFA in PBS and labeled using FITC-conjugated Bandeiaea simplicifolia lectin (Sigma-Aldrich) and Cy3-conjugated anti-α-smooth-muscle actin (αSMA) antibody (Sigma-Aldrich). Images were captured using an LSM 5 Pascal laser-scanning confocal microscope (Carl Zeiss, Cambridge, UK) and analyzed using Imaris 3D reconstruction software (Bitplane, Zurich, Switzerland) (27).

### Immunofluorescent staining and confocal microscopy

OCT-embedded tumor samples were sectioned (50–100 μm) and fixed using 4% PFA (Sigma-Aldrich) in PBS for 15 min before blocking-permeabilization with 5–10% goat serum (Invitrogen) and 0.1–0.3%Triton-X-100 (Sigma-Aldrich) for 1 h. Samples were incubated at 4**°**C overnight with primary antibodies (Supplemental Table S1) in blocking solution, followed by secondary antibodies where necessary, and mounted in Prolong Gold (Invitrogen). Sections were imaged by confocal microscopy as described above and analyzed with Imaris software (Bitplane). An average of 3 random images/section and 3 sections/mouse were analyzed.

### Flow cytometry

Ascitic fluids from WT and EC JAM-C-KO mice were analyzed by flow cytometry to characterize infiltration of different leukocyte subpopulations in the peritoneal tumor model. Briefly, ascite samples were incubated with anti-mouse CD16/32 to block Fc receptors, followed by incubation with fluorescently labeled primary antibodies (Supplemental Table S1). Samples were then washed and analyzed on a Dako Cyan flow cytometer (DakoCytomation, Glostrup, Denmark), and data were analyzed using FlowJo software (Tree Star, Ashland, OR, USA).

### Western blot

The protein expression of platelet-derived growth-factor receptor β (PDGFRβ), endomucin, and β-tubulin in murine tumor samples was analyzed by Western blot as described previously (8) using standard procedures.

### Protein analysis of ascitic fluid

The protein expression profile of mouse ascitic fluid was analyzed with the Angiogenesis Proteome Profiler Array kit (R&D Systems, Abingdon, UK) as per the manufacturer's instructions. Densitometry images of the blots were analyzed with ImageJ (U.S. National Institutes of Health, Bethesda, MD, USA). Expression of selected proteins (PDGF-AA and PDGF-BB) was also analyzed by ELISA using commercial kits (R&D Systems).

### *In vivo* vascular permeability assay

Tumor vascular leakage was analyzed by i.v. injection of 100 μl Hoechst dye (Sigma-Aldrich) in saline solution (4 μg/ml). Animals were euthanized 10 min after injection, and tumors were removed, embedded in OCT, and frozen. Next, 50-μm tumor sections were immunostained for VE-cadherin, imaged by confocal microscopy, and quantified for volume of vascular leakage in relation to the total vessel volume using Imaris image analysis software (Bitplane) as previously detailed (8, 31).

### Statistics

Data analysis was performed using GraphPad Prism 4 (GraphPad, San Diego, CA, USA). Results are expressed as means ± se unless stated otherwise. Statistical significance was assessed by unpaired *t* test and log-rank tests. Correlations were analyzed by Pearson's correlation coefficient. Values of *P* < 0.05 were considered significant.

## RESULTS

### JAM-C is expressed in the vasculature of tumors in human ovarian HGSC and in the ID8 mouse model of ovarian cancer

**Figure 1.**
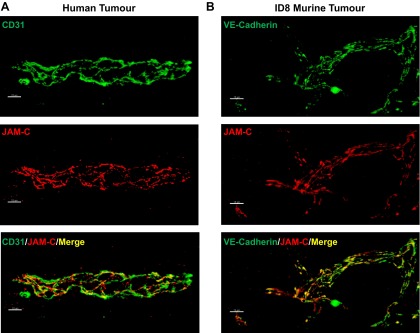
JAM-C is expressed in the tumor vasculature of both high-grade serous ovarian cancer and the ID8 murine model of ovarian cancer. Confocal microscopy images of sections from human (*A*) and mouse (*B*) ovarian tumors immunofluorescently stained for JAM-C (red) and the endothelial cell junctional markers CD31 or VE-cadherin (green). Images are representative of 4 human and 10 mouse samples. Scale bars = 10 μm.

Initial studies demonstrated the expression of JAM-C in both human and mouse ovarian cancer tumor vasculature. Human solid tumor tissues were obtained from patients with HGSC after cytoreductive surgery, and frozen sections were analyzed for JAM-C expression by immunofluorescence staining and confocal microscopy. Samples showed notable expression of JAM-C in blood vessels at EC junctions, as indicated through double staining of JAM-C and the EC junctional marker CD31 (**Fig. 1*A***). JAM-C was also detected in the vasculature of peritoneal ID8 tumors in mice. Similar to the human samples, in the murine model, tumor vessels expressed JAM-C at EC junctions, as indicated by expression of the EC junctional marker VE-cadherin (Fig. 1*B*). Collectively, these initial findings demonstrated that the vasculature of both human and mouse ovarian tumors expresses JAM-C on ECs.

### Endothelial JAM-C supports tumor growth in the murine ID8 model

**Figure 2.**
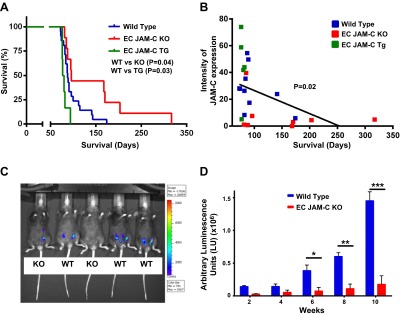
JAM-C expression affects tumor growth and survival in the ID8 model of ovarian cancer. *A*) Survival curves from WT, EC JAM-C-KO, and EC JAM-C-Tg mice following intraperitoneal injection of ID8 malignant cells (*n*=6–10 mice/group). *B*) Correlation of the expression of JAM-C in tumor vasculature and survival after ID8 cell injection. *C, D*) Tumor growth in WT and EC JAM-C-KO mice was monitored *in vivo* by measuring the bioluminescent intensity of ID8 cells. *C*) Representative image from the 10-wk time point. *D*) Analysis of the bioluminescent intensity over time (*n*=5 mice/group). *P < 0.05, **P < 0.01, ***P < 0.001.

We next sought to investigate the role of EC JAM-C in tumor growth in the ID8 mouse model of ovarian cancer. For this purpose, we used EC JAM-C-KO and EC JAM-C-Tg mouse colonies, mice that compared to WTs exhibit reduced (∼74%) and enhanced (∼123%) levels of JAM-C in their ECs, respectively, as analyzed by confocal microscopy (data not shown). Tumor development and the generation of ascitic fluid were monitored through abdominal swelling, and the animals were euthanized when ∼20% abdominal swelling was noted (commonly within a period of 12–15 wk) as per UKCCR guidelines (29). Initially, we compared the survival of WT mice in the ID8 model to that of EC JAM-C-KO and EC JAM-C-Tg mice. Similar to our previous findings (25), WT mice showed an endpoint of 88 d, but this was significantly enhanced in the EC JAM-C-KO mice (96 d, *P*=0.04) and reduced in the EC JAM-C-Tg mice (78.5 d, *P*=0.03) (**Fig. 2*A***). Of note, as no significant differences were observed between the average survival of WT control mice from the EC JAM-C-KO and EC JAM-C-Tg colonies (89 and 85.5 d, respectively), data acquired from the WT mice of these colonies were pooled.

Analysis of tumors in these animals showed a significant inverse correlation (*P*=0.02) between EC JAM-C expression levels in the tumor vasculature and survival outcome (Fig. 2*B*), providing additional supportive evidence to suggest a role for EC JAM-C in tumor development. To investigate this possibility directly, the rate of tumor growth over 10 wk was quantified in WT and EC JAM-C-KO mice implanted with luciferase-ID8 cells, enabling tumor size to be measured by luminescence (Fig. 2*C*). Using this assay, while WT mice showed an accelerated rate of tumor growth from 6 wk after injection of malignant cells, in EC JAM-C-KO mice, no significant tumor growth was noted during the 10-wk observation period (Fig. 2*D*). These results provide evidence for the ability of EC JAM-C to support the growth and aggressiveness of tumors in the ID8 ovarian cancer model.

### EC JAM-C does not support tumor leukocyte infiltration or vascularity in the ID8 model

**Figure 3.**
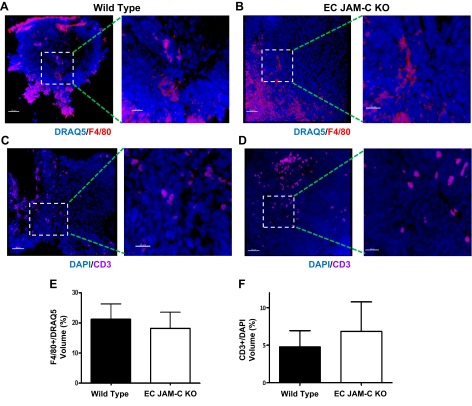
Immune cell infiltration into ovarian tumors is not affected by EC JAM-C expression. *A–D*) Representative confocal microscopy images of tumor sections from WT (*A*, *C*) and EC JAM-C-KO (*B*, *D*) mice immunofluorescently stained for macrophages (F4/80; *A*, *B*) or T cells (CD3; *C*, *D*). Nuclei are shown in blue. Right panels show enlarged views of boxed areas in left panels. Scale bars = 50 μm (left panels); 20 μm (right panels). *E*, *F*) Quantification of F4/80^+^ (*E*) and CD3^+^ (*F*) cells in tumor sections (*n*=5 mice/group).

To explore how EC JAM-C maybe regulating the size of ID8 tumors, we investigated the leukocyte infiltration profile and vascularity of tumors in EC JAM-C-KO mice as compared to WTs. With respect to the former, levels of T lymphocytes and macrophages, the primary immune cells recruited in this model (25), were quantified in tumor sections by immunofluorescent staining and confocal microscopy, as illustrated in **Fig. 3*A–D***. While both cell types were detected in tumors, no significant differences between the two mouse strains were observed (Fig. 3*E*, *F*). In addition, analysis of leukocyte infiltration in the ascitic fluid of the mice by flow cytometry showed no significant differences between the two strains in terms of percentage infiltration of B cells, T cells, monocytes, macrophages, neutrophils, or NK cells (data not shown).

**Figure 4.**
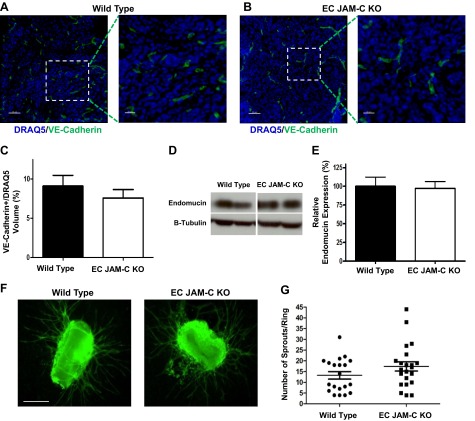
Tumor vessel formation is not affected by EC JAM-C expression. *A*, *B*) Representative confocal microscopy images of WT (*A*) and EC JAM-C KO (*B*) tumor sections immunofluorescently stained for VE-cadherin as a marker for ECs. Nuclei are shown in blue. Right panels show enlarged views of boxed areas in left panels. Scale bars = 50 μm (left panels); 20 μm (right panels). *C*) Quantification of the vessel volume in tumor sections from panels *A* and *B* (*n*=5 mice/group). *D*, *E*) Western blots (*D*) and densitometry quantification (*E*) of the relative levels of the EC marker endomucin in WT and JAM-C EC-KO tumor homogenates (*n*=7 mice/group). *F*) Representative images of an *ex vivo* aortic ring assay showing angiogenic sprouts formed in both WT and EC JAM-C-KO animals. *G*) Quantification of aortic ring sprouts from the images in panel *F* (*n*=20–22 rings from 4 mice/group).

To investigate tumor vessel density in WT and EC JAM-C-KO mice, tumor sections from both strains were analyzed for VE-cadherin expression by immunofluorescent staining and confocal microscopy (**Fig. 4*A–C***) or were measured for the EC protein endomucin by Western blot (Fig. 4*D*, *E*). Collectively, both methods showed comparable levels of tumor vascularity in WT and EC JAM-C-KO mice, suggesting that the delay on tumor growth in the EC JAM-C-KO mice was not due to reduced angiogenesis in these mice. This was confirmed *ex vivo* using an aortic ring assay model of angiogenesis, in which rings from WT and EC JAM-C-KO animals exhibited a similar angiogenic response (Fig. 4*F*, *G*).

### EC JAM-C is important in development of functionally viable tumor blood vessels

**Figure 5.**
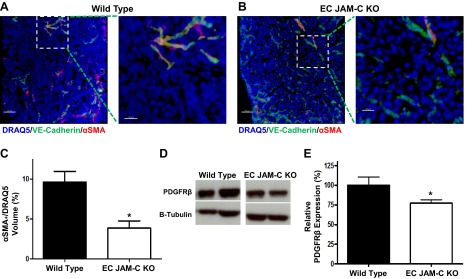
EC JAM-C expression affects pericyte coverage in the tumor vasculature. *A*, *B*) Representative confocal microscopy images of WT (*A*) and EC JAM-C-KO (*B*) tumor sections immunofluorescently stained for VE-cadherin (green) and αSMA (red) as markers for ECs and pericytes, respectively. Nuclei are shown in blue. Right panels show enlarged views of boxed areas in left panels. Scale bars = 50 μm (left panels); 20 μm (right panels). *C*) Pericyte coverage of the tumor vasculature in WT and EC JAM-C-KO mice was analyzed by measuring the percentage of αSMA colocalized with VE-cadherin from the images in panels *A* and *B* (*n*=5 mice/group). *D*, *E*) Western blots (*D*) and densitometry quantification (*E*) of the relative levels of the pericyte marker PDGFRβ in WT and JAM-C EC-KO tumor homogenates (*n*=7 mice/group). *P < 0.05.

**Figure 6.**
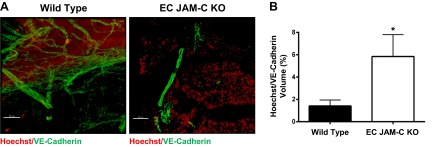
Deletion of EC JAM-C increased the permeability of the ovarian tumor vasculature. Permeability of the tumor vasculature was analyzed *in vivo*. The nuclear dye Hoechst was injected i.v. and left to circulate for 10 min before the animals were euthanized. Tumors were collected, sectioned, and immunofluorescently stained with the EC marker VE-cadherin. *A*) Representative images of tumor sections from WT and EC JAM-C-KO mice showing Hoechst (red) and VE-cadherin staining (green). Scale bars = 50 μm. *B*) Quantifications of the images in panel *A* showing the average extravascular Hoechst in relation to the vessel volume (*n*=5 mice/group). *P < 0.05.

Although no evidence was found for a notable change in tumor vessel density under conditions of EC JAM-C deletion, we hypothesized that perhaps the blood vessels formed in the KOs may be morphologically and/or functionally impaired. To address this possibility, we initially investigated the pericyte coverage of blood vessels using two different methods, measurement of α-SMA staining (colocalized with VE-cadherin) of tumor sections (**Fig. 5*A–C***) and analysis of tumors for the pericyte marker PDGFRβ by Western blot (Fig. 5*D*, *E*). Both approaches indicated that tumor blood vessels of EC JAM-C-KO mice show significantly reduced pericyte coverage as compared to WTs. Of note, RT-PCR analysis failed to detect mRNA expression of *PDGFR*β in ID8 cells (data not shown), indicating that the Western blot analysis of PDGFRβ in tumor samples was not influenced by potential expression of PDGFRβ in the tumor cells. As normal pericyte coverage is a key component of maintaining functionally viable blood vessels, we next investigated the permeability of tumor blood vessels in WT and EC JAM-C-KO mice as quantified by leakage of intravenously administered Hoechst dye into the tumor tissue. Results in **Fig. 6** show increased permeability in the tumor vasculature of EC JAM-C-KOs as compared to WT mice. Together, these results demonstrate that loss of EC JAM-C leads to development of morphologically and functionally abnormal tumor blood vessels that could account for reduced tumor growth in the present ID8 model of ovarian cancer.

## DISCUSSION

JAM-C is an adhesion molecule that can support leukocyte infiltration and angiogenesis (2). As these responses are key features of most cancers, in the present study we investigated the role of EC JAM-C in the development and pathogenesis of a murine model of ovarian cancer. Specifically, using genetically modified mice we provide evidence for the involvement of EC JAM-C in the growth and aggressiveness of peritoneal malignant tumors. Surprisingly, however, genetic deletion of EC JAM-C did not affect tumor leukocyte infiltration or extent of tumor vascularity but led to the formation of tumor blood vessels with defective pericyte coverage and compromised barrier function. The findings provide the first direct evidence for the involvement of EC JAM-C in development of experimental ovarian cancer and indicate that this could be accounted for by a previously unknown function of EC JAM-C as a regulator of morphologically and functionally viable blood vessels.

In initial studies, we found that JAM-C was expressed in the vasculature of human HGSC, an expression profile that was largely junctional, in line with the expected expression of JAM-C at EC tight junctions (2, 11). To investigate the functional role of EC JAM-C in ovarian cancer, a murine model involving intraperitoneal injection of ID8 cells was used. In this model, the ID8 cells lead to the formation of syngeneic ovarian tumors with many characteristics similar to those observed in advanced human ovarian cancers (24–26), and indeed the ID8 tumor vasculatures expressed JAM-C with a similar profile to that found in the human ovarian cancer samples. The importance of EC JAM-C in the development and aggressiveness of this model was directly demonstrated through the use of EC JAM-C-deficient and EC JAM-C-overexpressing mice, animals that showed a significantly prolonged and reduced survival in the ID8 model, respectively, as compared to WTs. Indeed, a significant inverse correlation was noted between expression of JAM-C in tumor vasculatures and survival, results that are in line with the almost total suppression of tumor development in EC JAM-C-deficient mice over a 10-wk period. Our findings are in agreement with recent studies that have also reported on the role of JAM-C as a mediator of tumor development and metastasis in other models (17–19).

In addressing the mechanism through which EC JAM-C supports tumor development, we investigated the effect of EC JAM-C deletion on the extent of tumor inflammatory cell infiltration and vascularity. Despite JAM-C being implicated in the pathogenesis of numerous acute and chronic inflammatory conditions (11, 12, 14), we observed no change in the tumor leukocyte infiltration or angiogenesis in EC JAM-C-deficient mice. To date, numerous studies have provided evidence for the involvement of JAM-C in regulation of leukocyte trafficking in a diverse range of inflammatory models (2, 11, 15). In addition, a JAM-C functional blocking antibody has been shown to suppress tumor growth and inhibit angiogenesis in a model of Lewis lung carcinoma (18), and cleaved soluble JAM-C can induce angiogenesis both *in vitro* and *in vivo* (10). The apparent lack of a functional role for EC JAM-C in leukocyte infiltration and angiogenesis in our ID8 model suggests that the role of JAM-C in regulation of EC functions may be governed by the model employed, as previously noted for numerous other EC junctional adhesion molecules (32). However, as our analysis of infiltration of leukocyte subpopulations was not exhaustive, a potential difference in recruitment of certain other leukocyte subpopulations not analyzed as part of this study, *e.g.*, regulatory T cells or M1 *vs.* M2 macrophages, between WT and EC JAM-C-deficient mice cannot be ruled out. Of note, there is also currently divergent evidence for the involvement of other JAM family members in angiogenesis; for example, JAM-A-deficient animals show reduced tumor vascularization in a model of pancreatic islet carcinoma (33), while JAM-B has been reported to have antiangiogenic properties with JAM-B-deficient mice showing increased tumor angiogenesis in a melanoma model (34).

Although normal vessel density was noted under conditions of EC JAM-C deletion in the ID8 tumor vasculatures, EC JAM-C-deficient mice developed tumor blood vessels with significantly suppressed pericyte coverage, as indicated by reduced staining for α-SMA and PDGFRβ. Furthermore, the tumor blood vessels of EC JAM-C-deficient mice were significantly leakier as compared to those in WT mice, collectively suggesting the existence of vascular dysfunction in the KO animals. There is now ample evidence associating pericyte coverage with normal vascular development and function (35), and inhibiting pericyte recruitment can affect the development of tumors. For example, PDGFR inhibitors have been shown to inhibit the growth of tumors through the detachment of pericytes from the vasculature (36), and the genetic deletion of pericytes through the use of a viral thymidine kinase slowed primary tumor growth (37). Furthermore, it has been proposed that the normalization of tumor vessels and the surrounding pericytes can help to improve the efficacy of cytotoxic therapy (38). Collectively the current evidence has raised much interest in the functional role of pericytes in maturation of tumor blood vessels, triggering enthusiasm for the potential benefits of targeting pericytes for future cancer therapy.

The mechanism through which EC JAM-C regulates pericyte recruitment remains unclear but may be associated with the promotion of a proangiogenic environment that would support migration of pericytes to newly formed blood vessels. However, in attempting to address this possibility, we found no differences in levels of key angiogenic factors (*e.g.*, VEGF, PDGF-aa, and PDGF-bb) in tumor peritoneal exudates from WT and EC JAM-C-deficient mice (Supplemental Fig. S1). Another possible mechanism may involve the ability of EC JAM-C to directly interact with a pericyte-associated ligand and, as such, support recruitment of pericytes to newly formed blood vessels. The principal JAM-C ligands are JAM-C, JAM-B, and the leukocyte integrin αMβ2 (Mac-1), but our findings to date have not provided any evidence for expression of JAM-C on tumor pericytes, and the potential expression of other JAM-C ligands on pericytes needs to be the subject of future studies.

Collectively, our results provide the first direct evidence for the involvement of EC JAM-C in the development and growth of peritoneal tumors as investigated in a murine model of ovarian cancer. The mechanism through which EC JAM-C supports tumor growth appears to be linked to the recruitment of pericytes to newly formed tumor blood vessels and the resultant development of a functionally stable tumor blood vasculature. This identifies a previously unknown role for EC JAM-C and strengthens the argument for further investigations into the potential role of EC JAM-C as a target for development of antitumor therapies.

## Supplementary Material

Supplemental Data
