# Supplementary material for: Endothelial cell junctional adhesion molecule C plays a key role in the development of tumors in a murine model of ovarian cancer
Source: FASEB J. 2013 Oct;27(10):4244–53. doi: 10.1096/fj.13-230441 (PMC3819510; doi:10.1096/fj.13-230441)
Supplement: Supplemental Data [file supp_27_10_4244__index.html]

Endothelial cell junctional adhesion molecule C plays a key role in the development of tumors in a murine model of ovarian cancer — Supplemental Data 

# Endothelial cell junctional adhesion molecule C plays a key role in the development of tumors in a murine model of ovarian cancer

## Supplemental Data

**Files in this Data Supplement:**

- Supplemental Data - (*13-230441SuppData.zip; compressed file 110 KB*)
