# Supplementary material for: Endothelial cell junctional adhesion molecule C plays a key role in the development of tumors in a murine model of ovarian cancer
Source: FASEB J. 2013 Oct;27(10):4244–53. doi: 10.1096/fj.13-230441 (PMC3819510; doi:10.1096/fj.13-230441)
Supplement: Supplemental Data [file supp_fj.13-230441_13-230441SuppData.zip › Supplementary Table 1.pdf]

Antigen	Species	Use	Clone	Source
**α-Smooth Muscle Actin**	Mouse	IF	1A4	Sigma Aldrich
**CD3e**	Rat	IF	17A2	eBioscience
**F4/80**	Rat	IF	Cl:A3-1	Abd Serotec
**VE-Cadherin**	Rat	IF	BV14	Ebioscience
**JAM-C (Murine)**	Rabbit	IF	322501	B.A. Imhof
**JAM-C (Human)**	Rabbit	IF	714	B.A. Imhof
**CD31**	Mouse	IF	JC70A	DAKO
**PDGFR-β**	Rabbit	WB	28E1	Cell Signalling
**Endomucin**	Rat	WB	V.7C7	Santa Cruz
**β-tubulin**	Mouse	WB	Tub 2.1	Sigma Aldrich
**Gr1**	Rat	FC	RB6-8C5	BD Pharmingen
**F4/80**	Rat	FC	BM8	Ebioscience
**B220**	Rat	FC	RA3-6B2	Biolegend
**CD115**	Rat	FC	AFS98	Ebioscience
**CD8**	Rat	FC	53-6.7	Ebioscience
**CD3**	Rat	FC	17A2	Ebioscience
**NK1.1**	Rat	FC	PK136	BD Pharmingen

Supplementary Table 1. List of Antibodies used for immunofluorescence staining (IF), Western blotting (WB) and flow cytometry (FC).
