# Supplementary material for: Endothelial cell junctional adhesion molecule C plays a key role in the development of tumors in a murine model of ovarian cancer
Source: FASEB J. 2013 Oct;27(10):4244–53. doi: 10.1096/fj.13-230441 (PMC3819510; doi:10.1096/fj.13-230441)
Supplement: Supplemental Data [file supp_fj.13-230441_13-230441SuppData.zip › Supplementary Figure 1.pdf]

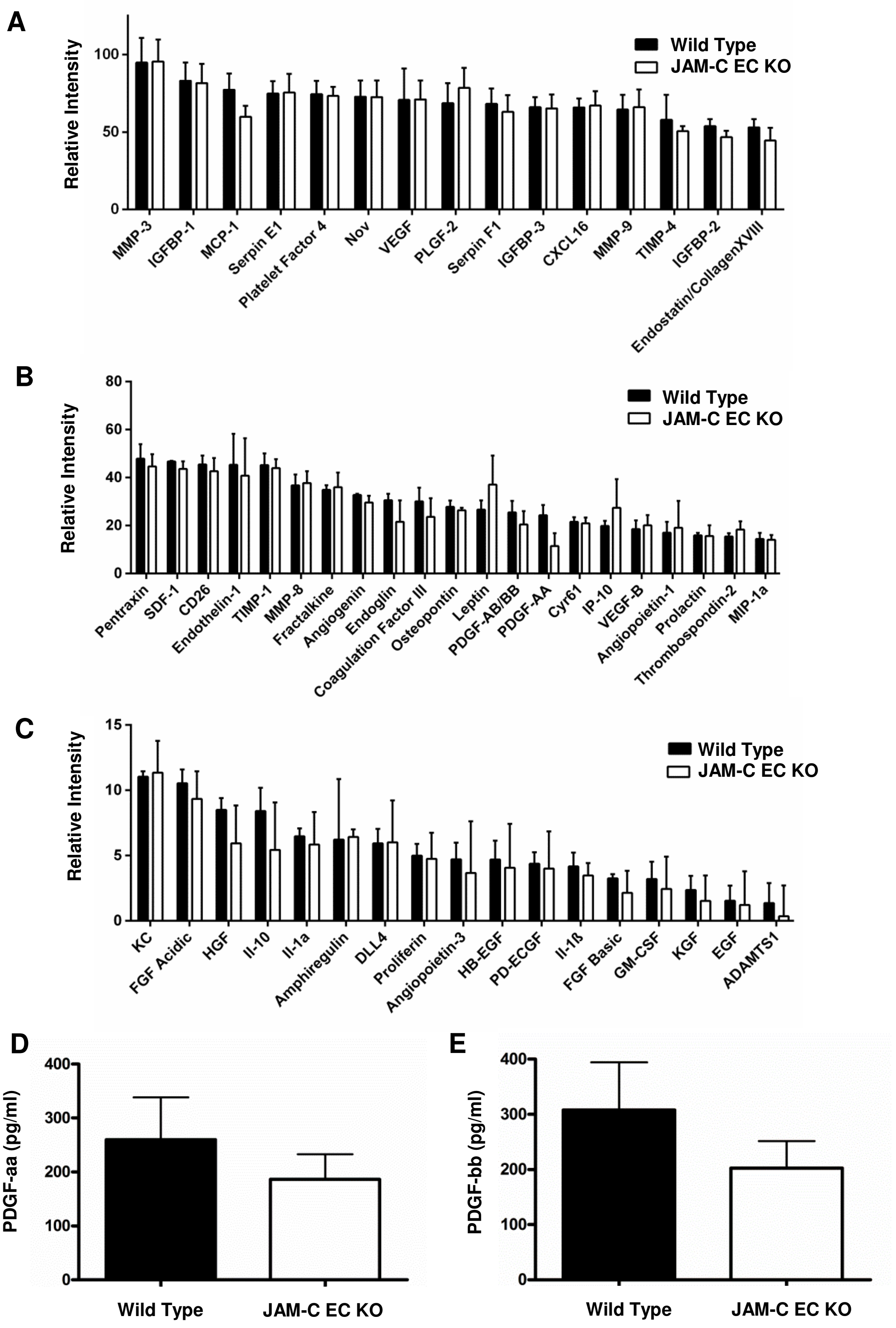

Supplementary Figure 1. Analysis of the angiogenic proteins present in the tumour ascitic fluid. (A-C) The relative levels of the indicated angiogenesis-related proteins were measured in ascitic fluid from wild type and JAM-C EC KO mice (n = 3 mice/group) with a comercial Proteome Array kit. The concentration of PDGF-AA (D) and PDGF-BB (E) in ascitic fluid was measured by ELISA (n = 9 mice/group).
